# Feeding ecology of fishes associated with artificial reefs in the northwest Gulf of Mexico

**DOI:** 10.1371/journal.pone.0203873

**Published:** 2018-10-02

**Authors:** Kaylan M. Dance, Jay R. Rooker, J. Brooke Shipley, Michael A. Dance, R. J. David Wells

**Affiliations:** 1 Department of Marine Biology, Texas A&M University at Galveston, Galveston, Texas, United States of America; 2 Department of Wildlife and Fisheries Sciences, Texas A&M University, College Station, Texas, United States of America; 3 Texas Parks and Wildlife Department, Artificial Reef Program, Austin, Texas, United States of America; 4 Department of Oceanography and Coastal Sciences, Louisiana State University, Baton Rouge, Louisiana, United States of America; Department of Agriculture and Water Resources, AUSTRALIA

## Abstract

The feeding ecology of two reef fishes associated with artificial reefs in the northwest Gulf of Mexico (GoM) was examined using gut contents and natural stable isotopes. Reefs were divided into three regions (east, central, west) across an east to west gradient of increasing reef complexity and salinity. Gray triggerfish (*Balistes capriscus*) primarily consumed reef-associated prey (xanthid crabs, bivalves, barnacles) and pelagic gastropods, while red snapper (*Lutjanus campechanus*) diets were mainly comprised of non-reef prey (stomatopods, fishes, portunid crabs). Natural stable isotopes of carbon (δ^13^C), nitrogen (δ^15^N), and sulfur (δ^34^S) were measured in consumer muscle tissue as well as potential primary producers. Gray triggerfish occupied a lower trophic position than red snapper, with lower δ^13^C and δ^15^N values across all size classes and regions, and generally higher δ^34^S values. Red snapper had a smaller range of stable isotope values and corrected standard ellipse areas across all size classes and regions, indicating a smaller isotopic niche. Contribution estimates of particulate organic matter (26 to 54%) and benthic microalgae (BMA, 47 to 74%) for both species were similar, with BMA contributions greater across all three size classes (juveniles, sub-adults, adults) of red snapper and all but the juvenile size class for gray triggerfish. Species gut contents and stable isotopes differed by region, with fishes consuming more crabs in the east region and more gastropods in the central and west regions. δ^13^C and δ^15^N values generally decreased from east to west, while δ^34^S increased across this gradient. Results highlight species-specific feeding differences associated with artificial reefs, where gray triggerfish may be more dependent on the reef structure for foraging opportunities. In addition, results offer further information on the integral role of BMA in primary production at nearshore artificial reefs.

## Introduction

Artificial reefs are frequently deployed in marine ecosystems to increase fisheries yields and enhance production of reef-associated fauna [1–3]. These goals are contingent on the premise that artificial reefs provide reef fishes and invertebrates with functionally similar habitat to natural reefs [4, 5]. While it is evident that high densities of economically and ecologically important species are often associated with artificial reefs [6, 7], their ecological role has continually been debated [8–12]. Nevertheless, global use of artificial reefs as fisheries management tools continues to increase [13, 14], and thus there is a need to further clarify the functional role artificial reefs provide to economically important species.

Studies investigating trophic interactions of faunal communities can provide useful data on sources of production and energy pathways [15–17]. Despite the use of artificial reefs by many economically valuable fishes [18, 19], our understanding of the feeding ecology of common predators associated with these structures remains limited [20]. Examination of predatory reef fish diets and trophic interactions, and identification of sources of primary production, is needed at artificial reefs to better understand their role as habitat to these species.

Conventional gut content analysis and natural stable isotopes have been used in combination to reconstruct feeding patterns and discern complex trophic interactions of faunal communities [21–23]. Gut content analysis is an indicator of recent (hours to days) feeding [24], and can be used to discern detailed predator-prey interactions and indicate potential competition interactions [25]. Stable isotopes of carbon (δ^13^C), nitrogen (δ^15^N), and sulfur (δ^34^S) provide a longer-term measure of diet, and are commonly used to determine trophic position and delineate trophic pathways [26–28]. δ^13^C values of predators reflect consumer diet and are useful for discerning contributions from different primary producers (e.g. pelagic vs. benthic), while δ^15^N can be used to estimate trophic position when baseline (from primary producers) δ^15^N values are known [27, 28]. Like δ^13^C, predator δ^34^S values can be used to distinguish between primary producers in systems where rates of sulfate reduction greatly differ, as is the case for seawater (higher in δ^34^S) and benthic sediment (lower in δ^34^S) [28]. Even though stable isotopes are commonly used to discern trophic interactions, this technique alone often lacks the resolution needed to reconstruct food webs and track energy flow [27]. Therefore, gut content analysis paired with stable isotopes results is a more integrative assessment of consumer feeding ecology than either method alone.

This study examined the feeding ecology of two reef fishes at artificial reefs in the northwest Gulf of Mexico (GoM), gray triggerfish (*Balistes capriscu*s) and red snapper (*Lutjanus campechanus*). These species are among the more abundant and frequently targeted fishes by recreational and commercial fisheries at artificial reefs in the GoM [29, 30]. While gray triggerfish and red snapper often co-occur on artificial reefs [31, 32], our understanding of their trophic interactions is lacking. Current knowledge on red snapper feeding ecology is primarily limited to the northeast and north-central GoM [23, 33–35], where biomass and fecundity estimates are lower compared to the northwest GoM. In addition, physicochemical and hydrographic conditions in the northeast and central GoM are considerably different from the northwest GoM [36, 37], which could result in differences in prey availability. Information on the trophic ecology of gray triggerfish at artificial reefs in the GoM is even more limited, focusing on predator-prey interactions with a single prey type (sand dollars) [38, 39]. The aim of this study was to use gut content analysis paired with natural stable isotopes to examine and contrast the role of artificial reefs as foraging habitat for these two reef-associated predators. In addition, regional feeding patterns were examined across an east to west coastal gradient to examine spatial variation in diet.

## Materials and methods

### Study area and sample collection

**Fig 1:**
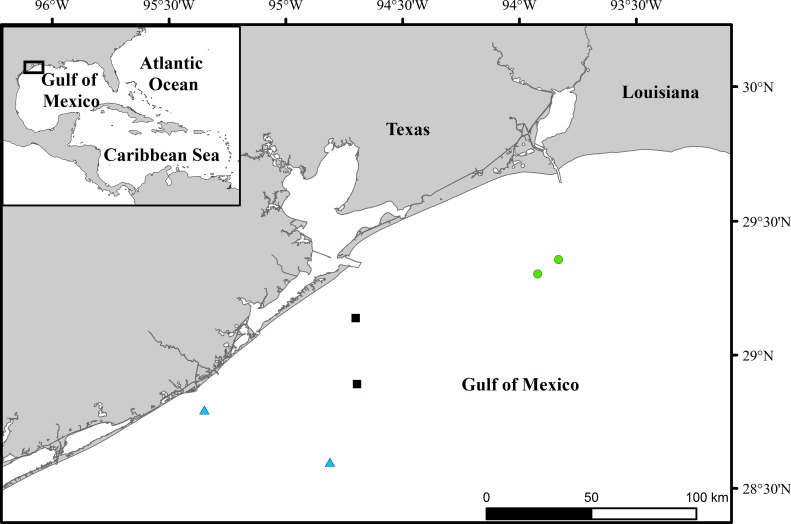
Location of study sites off the texas coast in the northwest Gulf of Mexico. Reefs were grouped into three regions, east (green circles), central (black squares) and west (blue triangles).

Sampling occurred from May to August of 2015 at nearshore (< 60 km from the shoreline) artificial reefs in the northwest GoM. Sites were distributed from east to west, and grouped into three regions (approximately 100 km apart; Fig 1). Salinity is lowest and freshwater inflow is highest in the east region, and subsequent increases in salinity and decreases in the rate of freshwater input occur into the central and west regions [40]. Reefs were located in depths ranging from 13 to 32 m, and were comprised of a variety of low-relief materials (< 3 m above seafloor) including quarry rocks, U.S. Department of Transportation Maritime Administration (MARAD) buoy pieces, concrete anchors, and reef pyramids in the east region. The central region was comprised of mid-relief (to 5 m above seafloor) quarry rocks, concrete blocks, culverts, reef balls, and disassembled platforms. The west region included high-relief (13 m above seafloor) sunken vessels, in addition to structures present in the east and central regions (concrete blocks, culverts, pyramids, and disassembled platforms). All sampling procedures in this study were approved by the Institutional Animal Care and Use Committee at Texas A&M University (Galveston Campus) and all efforts were made to minimize animal suffering during collection. Procedures were approved by and carried out under a permit issued by the Texas Parks and Wildlife Department (SPR-0314-050), as well as Letters of Acknowledgement from the National Marine Fisheries Service.

Reef sites were surveyed one to two times during the sampling period using two sampling gears to obtain a wide size range of gray triggerfish and red snapper at each site. Larger individuals were collected via standardized vertical longlines, using a protocol similar to the Southeast Area Monitoring and Assessment Program (SEAMAP) [41], while smaller individuals were targeted using traps. Sampling at each artificial reef site consisted of three sets of vertical longline and paired trap deployments (total of six traps) at three locations within the reef site. Each vertical longline set was comprised of four separate drops of a backbone (10 hooks), containing one of four hook sizes (2/0, 8/0, 11/0 and 15/0 Mustad circle hooks). Each hook size was fished for five minutes, while holding a fixed position over the reef. Oval fish traps (volume = 19,000 cm^3^, mesh size = 0.63 cm) were soaked for approximately one hour, and Atlantic mackerel (*Scomber scombrus*) was used as bait for both vertical longlines and traps.

Salinity, temperature, and dissolved oxygen were measured during each survey using a Hydrolab multiparameter sonde. In addition, particulate organic matter (POM) and benthic microalgae (BMA) were collected during surveys to measure stable isotope compositions of two primary sources (i.e., producers) of organic matter. Seawater was collected at each reef site, and POM was isolated by filtering seawater over precombusted (1 h at 450°C) 47 mm GF/F filters with a 0.7 μm pore size, and was used as a proxy for phytoplankton. Sediment was collected via a Ponar benthic grab (15.2 x 15.2 cm), from which BMA was isolated for stable isotope analysis following the vertical migration technique described by Wells et al. [23]. However, BMA was not collected in the west region due to multiple failed sampling attempts.

### Stable isotopes and gut content analysis

Fishes were immediately placed on ice in the field for transport back to the laboratory, where they were stored at -20°C until processing. Fishes were weighed to the nearest g and measured to the nearest mm total length (TL) and fork length (FL). The stomach and intestinal tract (gut) were removed from each individual, weighed to the nearest g, incised, fixed in 10% formalin for 24 to 48 hours, and then preserved in 70% ethanol until gut content analysis was performed. Gut contents were sorted, enumerated, and identified to the lowest possible taxon, and subsequently dried at 60°C for 24 h and weighed to the nearest 0.0001 g.

Epaxial muscle tissue was taken from gray triggerfish and red snapper (left side of the fish) and dried for 24 h at 60°C. Each tissue sample was lipid-extracted using an Accelerated Solvent Extractor (Model 300) by Dionex, as described by Plumlee and Wells [42], and homogenized with a ball and mill grinder (Wiggle-Bug^®^). A subsample of the resultant powder for each individual sample was then weighed (0.8 to 1.2 mg) and packaged into tin capsules. Dried filters with POM and BMA were cut in half, and edges not containing sample material were removed. Half of the filter was then weighed to the nearest mg and packaged into a tin capsule for analysis. Natural stable isotope values of δ^13^C, δ^15^N, and δ^34^S were determined using an elemental analyzer interfaced to a continuous flow isotope ratio mass spectrometer (IRMS) at the University of California-Davis Stable Isotope Facility. δ^34^S values were not obtained for POM and BMA due to collection on filters, which resulted in compromised δ^34^S values. Stable isotope values are reported in delta notation relative to Vienna PeeDee belemnite for carbon, atmospheric nitrogen (N_2_) for nitrogen, and Vienna Canyon Diablo troilite for sulfur using the following equation: where *R* represents the ratio of heavy to light isotopes (^13^C/^12^C, ^15^N/^14^N, ^34^S/^32^S).

$$\delta^{13}C,\;\delta^{15}N\;\mathrm{or}\;\delta^{34}S\left(‰\right)=\left(\frac{R_{\mathrm{sample}}}{R_{\mathrm{standard}}}‑1\right)x\;1000$$

### Data analysis

Gray triggerfish and red snapper feeding was examined across three size classes based on size-at-age models for gray triggerfish [43] and red snapper [44]. Size classes, representing approximate life-stages, consisted of juveniles [age 0–1, gray triggerfish: 111 to 183 mm FL (mean ± SE = 141.92 ± 7.35 mm), red snapper: 145 to 222 mm FL (190.39 ± 2.43 mm)], sub-adults [age 2–3, gray triggerfish: 183 to 283 mm FL (232.82 ± 3.84 mm), red snapper: 223 to 356 mm FL (297.66 ± 2.64)], and adults [age 4 +, gray triggerfish: 284 to 382 mm FL (318.24 ± 4.44 mm), red snapper: 357 to 570 mm FL (422.67 ± 5.67 mm)]. While the majority of individuals are sexually mature by age 4 [45, 46], individuals of both species can reach sexual maturity as early as age 2 [45, 47], thus the sub-adult size class likely included both immature and mature fishes. Because reef structure was inconsistent among regions, observed differences in regional gut contents or stable isotopes could not be attributed solely to reef material or region. Significance was determined at an alpha value of 0.05 for all statistical analyses, and all measures of error are standard error (SE) unless indicated otherwise.

#### Gut contents

Gut content analyses were performed on a total of 89 gray triggerfish and 259 red snapper (Table 1). Empty guts and those solely containing unidentifiable content, chyme, bait, parasites, and inorganic material (rocks, plastic, lures) were excluded from the analysis (2% of gray triggerfish and 21% of red snapper). Identifiable contents were then categorized into 16 taxonomic groups. Several prey groups comprised less than 1% of the total dry weight (amphipods, bryozoans, echinoderms, isopods, polychaetes, sargassum, shrimp, squid, and zooplankton), thus quantitative analysis was restricted to the 7 most common prey groups: barnacles, bivalves, cnidarians, crabs, fish, gastropods, and stomatopods. Percent frequency of occurrence (%FO), percent composition by number (%N), and percent composition by dry weight (%W) were computed for each prey group. Likewise, a percent index of relative importance (%IRI) was calculated to integrate both weight and numerically based measures (%FO, %N) of diet following the equation by Pinkas et al. [48]. The %W of dominant prey groups was used as the dependent variable for all statistical analyses of diet, as it is a useful proxy for estimating the nutritional contribution of prey groups [24]. Prey contributions to species’ diets were estimated from maximum likelihood estimates using a diet mixture model described by Moriarty et al. [49]. Because this model assumes that the prey group for which contributions are being estimated makes up 100% (%W) of the contents for a minimum of one consumer’s gut (where the proportion (p) = 1), a reduced model (where this probability was assumed to be 0 instead of 1) was used when none of the samples met this assumption [49]. Percent composition by weight was square-root transformed to reduce the importance of dominant prey groups and used to create a Bray-Curtis similarity matrix. Permutational analysis of variance (PERMANOVA) and a posteriori tests were then conducted on the resulting matrix to assess the effect of size class, species, and region on prey group composition in PRIMER v.7 [50]. Similarity percentages (SIMPER) were then used to identify prey groups with the greatest contribution to the dissimilarity among size classes, between species, and among regions. Furthermore, the %W of family-level taxa comprising the 5 (of 7) prey groups identified by SIMPER were examined to assess differences in taxa within these prey groups.

**Table 1:** Gray triggerfish and red snapper sample sizes for gut content and stable isotope analyses.

	Gut contents			Stable isotopes				
	East	Central	West	Total	East	Central	West	Total
**Gray triggerfish**								
Juveniles	3	2	8	**13**	3	2	7	**12**
Sub-adults	19	22	3	**44**	20	21	3	**44**
Adults	9	8	15	**32**	9	8	16	**33**
**Red snapper**								
Juveniles	25	12	15	**52**	28	22	19	**69**
Sub-adults	99	36	9	**144**	115	46	15	**176**
Adults	54	7	2	**63**	60	15	7	**82**

Sample sizes are shown by size class, species, and region. Gut content and stable isotope analyses were conducted on the same individuals, where empty guts and those solely containing unidentifiable content, chyme, bait, parasites, and inorganic material (rocks, plastic, and lures) were excluded from the analysis.

#### Stable isotopes

Multivariate analysis of variance (MANOVA) was used to test for differences in δ^13^C, δ^15^N, and δ^34^S stable isotope values among size classes, between species, and among regions for gray triggerfish and red snapper. δ^13^C, δ^15^N, and δ^34^S were included as dependent variables in a three factor MANOVA with size class, species, and region as independent variables. Regional differences in δ^13^C and δ^15^N values between sources, POM [east (n = 3), central (n = 6), west (n = 3)] and BMA [east (n = 3), central (n = 6)], were also examined using MANOVA, with δ^13^C, δ^15^N, and δ^34^S as dependent variables and source and region as the independent variables. The influence of each independent variable was then examined for each dependent variable (δ^13^C, δ^15^N, and δ^34^S) using a one-way analysis of variance (ANOVA). Pairwise differences among means were examined using Shaffer’s multiple comparison procedure (Shaffer’s MCP) [51, 52], as it is less affected by unbalanced sample sizes than other post-hoc tests and still controls for Type I error. Statistical analyses were performed in R [53] using the multcomp package [54].

#### Isotopic niches

Standard ellipse areas (SEA) and niche metrics, including the mean distance to centroid and stable isotope ranges [55–57], were computed using Stable Isotope Bayesian Ellipses in R (SIBER). Analyses were performed for each species by size class and region (with size classes combined), as multiple size classes (when examined within region) for both species did not meet the minimum (n = 10) recommended sample size for reliable niche width estimates [56]. Because the regional analysis encompassed multiple size classes, values of δ^13^C, δ^15^N and δ^34^S were length adjusted according to the following equation [58] to account for isotopic relationships with size [59], where δ*X*ʹ = adjusted isotope values, δ*X* = raw isotope value, *a* = regression coefficient, and FL = fork length of fish (mm).

$$\delta X^{\prime }=\delta X-(a\;x\;FL)$$

SEA, representing a group’s core isotopic niche, was calculated for each species, size class, and region. To minimize bias due to small sample size, SEA was subsequently corrected to SEA_c_ [57], and then used to calculate potential isotopic niche overlap. Overlap between ellipses was considered significant when greater or equal to 0.60, representing 60% overlap between two group’s SEA_c_’ [60, 61]. Credible intervals were then obtained for isotopic niche areas for statistical comparison using a Bayesian technique detailed by Jackson et al. [56]. In addition, niche metrics were calculated based on the individuals used to determine isotopic niche areas. The mean distance to centroid (CD) serves as a measure of group trophic diversity, while nitrogen range (NR), carbon range (CR) and sulfur range (SR) represent the ranges of δ^13^C, δ^15^N and δ^34^S exhibited by each species [55]. Sample size varied for size classes, species, and regions, thus niche metrics (CD, NR, CR, SR) were bootstrapped (n = 10,000) based on the group with the smallest sample size for statistical comparison based on resultant confidence intervals.

#### Source contributions

Relative contributions of pelagic (POM) and benthic (BMA) carbon to the diets of juvenile, sub-adult, and adult gray triggerfish and red snapper were estimated using Bayesian mixing models in MixSIAR [62]. Individual species stable isotope values were used with trophic discrimination factors of 1.3 ± 0.30 ‰ SD for δ^13^C, and 3.4 ± 0.60 ‰ SD for δ^15^N [63, 64]. Trophic level for each individual, which was then used to estimate the average trophic level for each species and size class, was calculated according to Post [27]:

$$Trophic\;level=1+(\delta^{15}N_{fish}-\delta^{15}N_{prod})/\Delta_{n}$$

where δ^15^N_fish_ is the δ^15^N value of an individual consumer (gray triggerfish or red snapper), δ^15^N_prod_ is the mean δ^15^N value of the primary producers [POM (n = 12), BMA (n = 9)], and Δ_n_ is the trophic discrimination factor for each trophic level. Primary producer stable isotope values were pooled across regions for use in the source contribution models, as δ^13^C and δ^15^N values did not significantly differ by region (F_4, 32_ = 146.23, p = 0.702, MANOVA). Models in MixSIAR were not concentration dependent, and comprised both residual and process error with 100,000 (50,000 burn-ins) iterations for all gray triggerfish size classes and juvenile red snapper. Models for sub-adult and adult red snapper were comprised of 300,000 iterations (200,000 burn-ins) due to failure to converge using 100,000 (50,000 burn-ins) iterations. To verify model convergence, Gelman-Rubin diagnostics were used [65]. Source contribution models for each species by region were not conducted due to insufficient sampling for POM and BMA within regions, where BMA was not collected in the west region.

## Results

Water parameters (salinity, temperature, and dissolved oxygen) were similar across the three regions (F_6, 20_ = 1.690, p = 0.175, MANOVA). Though not statistically different, salinity was lowest in the east (34.07 ± 0.76), and progressively increased in the central (35.78 ± 0.61) and west (36.07 ± 0.95) regions. Similarly, mean temperatures in the east, central and west regions were 28.19 ± 1.04°C, 29.19 ± 0.61°C, and 27.73 ± 1.12°C, respectively. Dissolved oxygen was 7.37 ± 0.62 mg l-1, 6.84 ± 0.51 mg l-1, and 7.10 ± 0.79 mg l-1, listed east to west. δ^13^C and δ^15^N values for primary producers (POM, BMA) did not significantly differ by region (F_4, 32_ = 146.23, p = 0.702, MANOVA), thus regional POM and BMA δ^13^C and δ^15^N values were pooled for use in the source contribution models. Primary producer δ^13^C values were significantly lower for POM (-22.50 ± 0.13 ‰) relative to BMA (-18.80 ± 0.20 ‰) (df = 19, p < 0.0001, Student’s t-test), while δ^15^N values were similar, 6.03 ± 0.31 ‰ for POM and 5.27 ± 0.35 ‰ for BMA (df = 19, p = 0.122, Student’s t-test). Due to failed sampling attempts, BMA was not collected from the west region. However, BMA stable isotope values did not differ between the east and central regions, and were comparable to previous reports throughout the GoM [15, 66]. Thus, BMA stable isotope values in the west region were assumed to be comparable to what was found for the east and central regions.

### Gut contents

**Fig 2:**
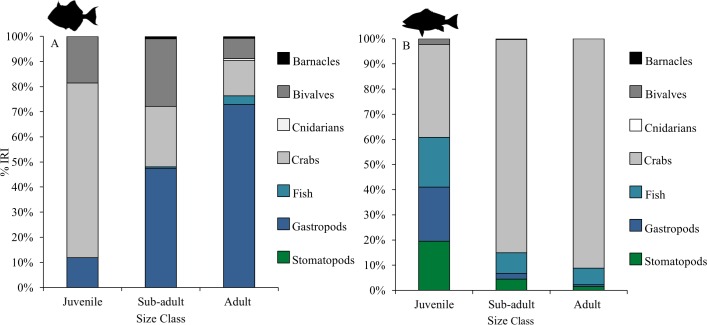
Percent index of relative importance (%IRI) for gray triggerfish and red snapper by size class. Prey groups shown are those accounting for more than 1% of the total percent weight (%W) in the gut contents. (A) Shows the %IRI for juvenile (n = 13), sub-adult (n = 44), and adult (n = 32) gray triggerfish gut contents, while (B) shows the %IRI for juvenile (n = 52), sub-adult (n = 44), and adult (n = 32) red snapper gut contents.

A total of 66 prey taxa in gray triggerfish and 47 in red snapper were identified, which were grouped into 7 primary prey groups (barnacles, bivalves, cnidarians, crabs, fish, gastropods, and stomatopods) for statistical analysis. Prey groups were similar among size classes (juveniles, sub-adults, adults) within each species, with the exception of juvenile gray triggerfish, which consumed a greater amount of crabs (predominately xanthid crabs) than sub-adult and adult fish (Table 2, Fig 2A, and S2 Table). However, the interpretation of diets quantified by bulk (%W) was greater influenced by the presence of unusual prey items, digestion rate, and order of ingestion at smaller sample sizes, which was considerably smaller for juvenile gray triggerfish (n = 13) compared to sub-adults and adults (Table 1) [51]. Thus, it is possible that the diet of juvenile gray triggerfish was not fully represented in the sample. Lastly, interaction terms pertaining to size class and region (size class x region, and species x size class x region) were non-significant (S1 Table). Thus post-hoc analyses of regional gut contents were carried out irrespective of size class for each species.

**Table 2:** Permutational analysis of variance (PERMANOVA) and similarity percentages (SIMPER) examining gut content composition.

PERMANOVA pair-wise tests	t	p-value	Unique perms	Dissimilarity (%)
**Size Class**				
*Gray Triggerfish*				
Juveniles, sub-adults	1.709	0.036*	999	60.72
Juveniles, adults	2.188	0.001*	997	72.50
Sub-adults, adults	0.523	0.915	999	NA
*Red Snapper*				
Juveniles, sub-adults	1.404	0.122	999	NA
Juveniles, adults	1.372	0.113	998	NA
Sub-adults, adults	0.114	1.363	999	NA
**Species**				
*Size class*				
Juveniles	2.686	0.001*	999	69.44
Sub-adults	2.770	0.001*	999	74.54
Adults	2.137	0.003*	998	77.71
*Region*				
East	2.727	0.001*	998	63.35
Central	2.968	0.001*	999	84.37
West	1.887	0.004*	998	82.43
**Region**				
*Gray triggerfish*				
East, Central	1.948	0.009*	999	64.51
East, West	1.834	0.01*	998	74.06
Central, West	1.233	0.193	999	NA
*Red Snapper*				
East, Central	4.800	0.001*	999	71.74
East, West	3.984	0.001*	999	83.80
Central, West	2.198	0.001*	999	84.10

Results from a posteriori tests following PERMANOVA examining prey group composition by size class, species, and region; a ‘*’ indicates a significant result. Percent dissimilarity between groups is additionally shown from similarity percentages (SIMPER). An ‘NA’ is shown where SIMPER was not applied due to non-significant differences between two groups.

**Fig 3:**
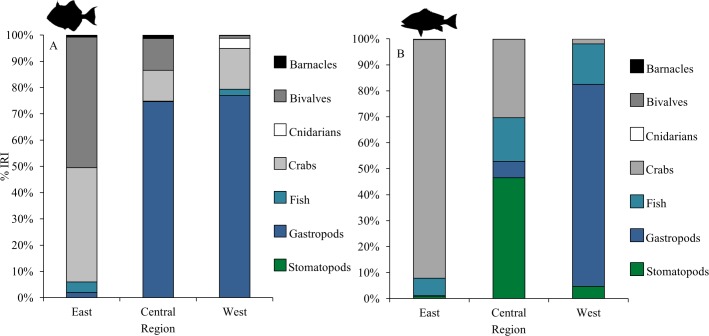
Percent index of relative importance (%IRI) for gray triggerfish and red snapper by region. Prey groups shown are those accounting for more than 1% of the total percent weight (%W) for (A) gray triggerfish in the east (n = 31), central (n = 32), and west regions (n = 26), and for (B) red snapper in the east (n = 178), central (n = 55), and west (n = 26) regions.

Species-specific differences in gray triggerfish and red snapper gut contents were consistent across all size classes and regions (Table 2). Prey groups (identified by SIMPER) most responsible for differentiation between gray triggerfish and red snapper diets were crabs, fishes, bivalves, gastropods, and stomatopods. Gray triggerfish %IRI and % contribution estimates from the diet mixture models for barnacles (exclusive to gray triggerfish gut contents), bivalves, and gastropods were greater for gray triggerfish than for red snapper across all size classes and regions, with the exception of fish in the west region, where gastropod %IRI and % contribution estimates were similar between species (Table 3, Figs 2 and 3). In contrast, red snapper %IRI and % contributions for stomatopods were consistently higher than those for gray triggerfish, as stomatopods were only present within adult gray triggerfish in the east region, where they contributed little to the diet (Table 3, Figs 2A and 3A). Red snapper %IRI and % contribution estimates for fish were also greater than those for gray triggerfish across most size classes and regions, with exception of adults, for which %IRI and % contribution estimates were similar between species (13.03 ± 4.16% for gray triggerfish and 11.29 ± 2.91% for red snapper) (Fig 2). The %IRI and % contribution estimates for crabs were generally higher for red snapper across all size classes and regions, with exception of juvenile gray triggerfish and gray triggerfish in the west region (Table 3 and Fig 2). The %W of family level taxa comprising primary prey groups identified by SIMPER varied by species, with gray triggerfish consuming more xanthid crabs and red snapper consuming more portunid crabs across all size classes and regions (S2 and S3 Tables). Likewise, gray triggerfish diets consisted of a larger percentage of pelagic gastropods from the family Cavolinidae across most size classes (Cavolinidae was not recorded in juvenile gray triggerfish) and all regions compared to red snapper (S2 and S3 Tables).

**Table 3:** Contribution estimates from the diet mixture models for each size class, species, and region.

	Size Class			Region		
Prey	Juveniles	Sub-adults	Adults	East	Central	West
**Gray Triggerfish**						
Barnacles	X	4.79 ± 1.75*	4.75 ± 1.65*	2.91 ± 1.5*	7.14 ± 2.05*	2.49 ± 1.8*
Bivalves	6.38 ± 2.84*	20.09 ± 3.73*	10.36 ± 3.49*	NA	17.8 ± 3.82*	2.26 ± 1.37*
Cnidarians	NA	0.47 ± 0.61	7.28 ±3.74*	NA	X	16.06 ± 6.24*
Crabs	43.24 ± 8.99	23.16 ± 4.04*	18 ± 4.15*	26.45 ± 4.42*	21.01 ± 4.46*	19.84 ± 6.5*
Fish	X	2.44 ± 0.94*	13.03 ± 4.16*	7.07 ± 2.68*	2.07 ± 0.94*	12.94 ± 6.19*
Gastropods	1.41 ± 1.27*	15.39 ± 4.17*	14.40 ±4.14*	1.36 ± 0.01*	30.63 ± 0.13*	9.23 ± 3.67*
Stomatopods	NA	NA	X	X	NA	NA
**Red Snapper**						
Barnacles	NA	NA	NA	NA	NA	NA
Bivalves	2.42 ± 1.32*	2.01 ± 1.13	X	1.05 ± 0.49*	0.63 ± 0.67*	X
Cnidarians	X	0.14 ± 0.14*	NA	0.07 ± 0.06*	NA	NA
Crabs	24.6 ± 6.06	38.51 ± 3.8	39.51 ± 4.23	45.49 ± 3.01	12.76 ± 4.15	4.70 ± 3.36
Fish	12.94 ± 4.34	12.14 ± 2.42	11.29 ± 2.91	10.44 ± 1.79	12.84 ± 4.63	16.05 ± 8.01
Gastropods	1.29 ± 5.97*	2.02 ± 0.94	0.63 ± 0.42*	0.13 ± 0.06*	1.45 ± 0.9	9.63 ± 4.6*
Stomatopods	24.7 ± 7.12	14.83 ± 3.48	6.47 ± 2.26	0.07 ± 0.02*	40.03 ± 7.44	18.91 ± 0.05

Values shown are prey group % contribution estimates along with the standard error for each size class, species, and region. An ‘X’ indicates an insufficient number of guts containing the prey item within the sample, where the model could not be applied. An ‘NA’ signifies that the prey item was not present in the sample, and a ‘*’ indicates results obtained using the reduced model.

Analysis of gray triggerfish and red snapper gut contents indicated differences in primary prey groups among regions (Table 2). Gray triggerfish gut contents in the east region significantly differed from those in the central and west regions (p < 0.01, PERMANOVA). This was additionally observed when examining gray triggerfish %IRI and % contribution estimates for gastropods, which were much greater in the central (30.63 ± 0.13%) and west (9.23 ± 3.67%) regions compared to the east region (1.36 ± 0.01%) (Fig 3A). Red snapper gut contents significantly differed among all regions (east, central, west), with crabs, stomatopods, fishes, and gastropods driving regional differences (according to SIMPER) (p < 0.05, PERMANOVA). Crab consumption (based on %IRI and % contribution estimates) was greatest in the east region (45.49 ± 3.01%), and declined in the central (12.76 ± 4.15%) and west (4.70 ± 3.36%) regions (Fig 3B). In contrast, red snapper %IRI and % contribution estimates for stomatopods were highest in the central region (40.03 ± 7.44%), while red snapper gastropod consumption (based of %IRI and % contributions) was greatest in the west region (Table 3, Fig 3B). Finally, both species %IRI and % contribution estimates for crabs were highest in the east region, while gastropod consumption (primarily pelagic taxa, Cavolinidae) increased in the central and west regions (Table 3, Fig 3, S3 Table).

### Stable isotopes

**Fig 4:**
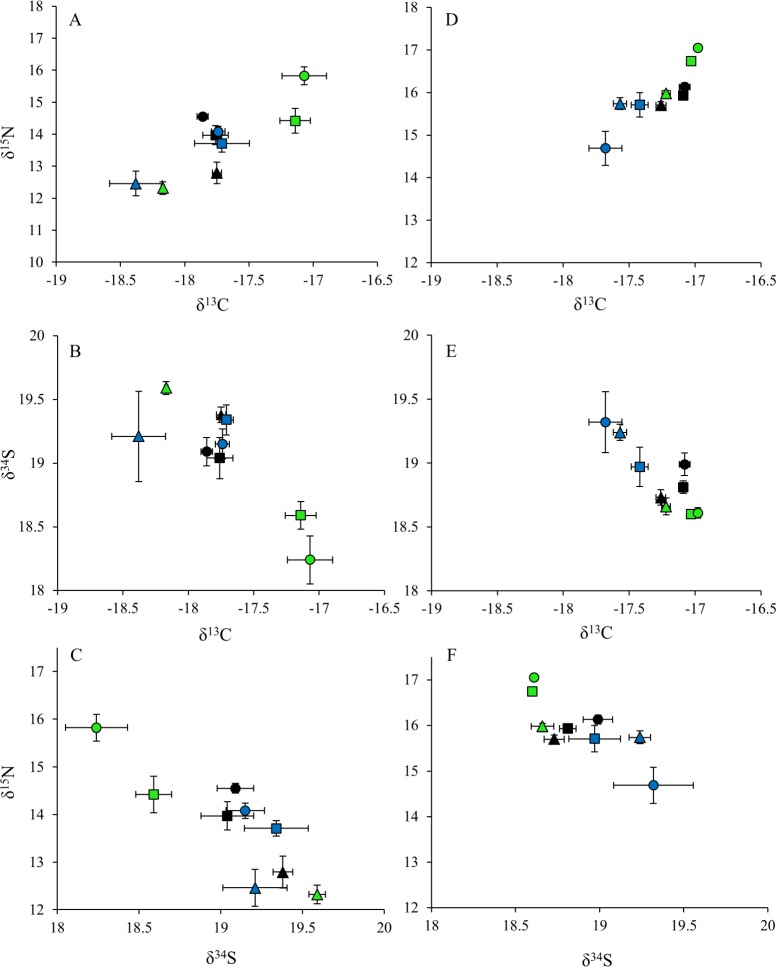
Mean stable isotope bi-plots for gray triggerfish and red snapper by size class and region. Panels A, B, and C (left) represent gray triggerfish, while panels D, E, and F represent red snapper (right). Means for δ^13^C, δ^15^N, and δ^34^S are shown for each species by size class, juveniles (triangles), sub-adults (squares), and adults (circles), and by region, east (green), central (black) and west (blue); error bars represent the standard error.

Gray triggerfish and red snapper δ^13^C and δ^15^N values generally increased with size across all regions (east, central, west), while δ^34^S values were similar across all size classes and regions, with the exception of juvenile fishes having higher δ^34^S values in the east region for gray triggerfish, and in the central region for red snapper (Fig 4, Table 4). Juvenile gray triggerfish δ^13^C values were significantly lower than sub-adult and adult fish within the east and west regions (p < 0.01, Shaffer’s MCP), and the same trend was observed for red snapper in the east and central regions (p < 0.01, Shaffer’s MCP). No differences in δ^13^C values were observed between size classes for gray triggerfish in the central region or for red snapper in the west region (Table 4, Fig 4). Though gray triggerfish δ^15^N values generally increased among juveniles, sub-adults and adults, δ^15^N values were only significantly lower for juveniles (12.32 ± 0.12 ‰) in the east region (p < 0.01, Shaffer’s MCP) (Table 4). Red snapper δ^15^N values increased significantly from juvenile to sub-adult size classes across all regions (p < 0.05, Shaffer’s MCP), with the exception of the west region, where δ^15^N values were similar among size classes (Table 4). Similarly, red snapper δ^15^N values increased between sub-adult and adult size classes in the east region (p < 0.0001, Shaffer’s MCP), but significantly decreased between sub-adult and adult size classes in the west region (p = 0.036, Shaffer’s MCP) (Fig 4C and 4F). Gray triggerfish δ^34^S values were significantly higher in juveniles compared to sub-adult and adult size classes in the east region (p < 0.01, Shaffer’s MCP), while juvenile red snapper δ^34^S values were significantly higher than adult fish in the central region (Table 4, Fig 4B and 4E) (p = 0.048, Shaffer’s MCP). Regional sample sizes for size class were low for gray triggerfish (Table 1), thus larger sample sizes may have resulted in additional differences in δ^13^C, δ^15^N, and δ^34^S among size classes within each region.

**Table 4:** Mean stable isotope values for δ^13^C, δ^15^N, and δ^34^S by size class, species, and region.

Group	δ^13^C	δ^15^N	δ^34^S
*Gray triggerfish*			
**East**			
Juveniles	-18.17 ± 0.01	12.32 ± 0.12	19.59 ± 0.05
Sub-adults	-17.14 ± 0.12	14.42 ± 0.28	18.59 ± 0.11
Adults	-17.07 ± 0.17	15.82 ± 0.28	18.24 ± 0.19
**Central**			
Juveniles	-17.75 ± 0.04	12.79 ± 0.34	19.38 ± 0.06
Sub-adults	-17.76 ± 0.1	13.97 ± 0.1	19.04 ± 0.16
Adults	-17.86 ± 0.04	14.55 ± 0.04	19.09 ± 0.11
**West**			
Juveniles	-18.38 ± 0.21	12.46 ± 0.39	19.21 ± 0.39
Sub-adults	-17.71 ± 0.21	13.71 ± 0.27	19.34 ± 0.27
Adults	-17.74 ± 0.53	14.08 ± 0.05	19.15 ±0.12
*Red snapper*			
**East**			
Juveniles	-17.22 ± 0.03	15.98 ± 0.07	18.66 ± 0.07
Sub-adults	-17.03 ± 0.02	16.74 ± 0.03	18.6 ± 0.04
Adults	-16.98 ± 0.02	17.05 ± 0.02	18.61 ± 0.04
**Central**			
Juveniles	-17.26 ± 0.04	15.7 ± 0.09	18.73 ± 0.06
Sub-adults	-17.09 ± 0.03	15.93 ± 0.06	18.81 ± 0.05
Adults	-17.08 ± 0.04	16.13 ± 0.1	18.99 ± 0.09
**West**			
Juveniles	-17.57 ± 0.05	15.74 ± 0.14	19.24 ± 0.06
Sub-adults	-17.42 ± 0.07	15.71 ± 0.29	18.97 ± 0.15
Adults	-17.68 ± 0.12	14.69 ± 0.4	19.32 ± 0.24

The mean ± SE (standard error) is shown for gray triggerfish and red snapper δ^13^C, δ^15^N, and δ^34^S values by size class, species, and region.

Gray triggerfish had generally lower δ^13^C and δ^15^N values than red snapper across all size classes and regions, while gray triggerfish δ^34^S values were generally higher (Table 4, S4 and S5 Tables). The sole exception to this was in the east region, where δ^34^S values were similar between species for sub-adults (p = 0.923, Shaffer’s MCP) and lower for adult gray triggerfish (p = 0.005, Shaffer’s MCP). Juvenile gray triggerfish δ^13^C values were significantly lower than those for juvenile red snapper across all regions (p < 0.001, Shaffer’s MCP), and sub-adult and adult gray triggerfish had significantly lower δ^13^C values than sub-adult and adult red snapper in the central region (p < 0.0001, Shaffer’s MCP). Though non-significant, sub-adult and adult gray triggerfish in the east and west regions also had lower δ^13^C values compared to red snapper within the same size classes (Table 4). Gray triggerfish δ^15^N values were significantly lower than those for red snapper across all size classes and regions (p < 0.0001, Shaffer’s MCP). Juvenile gray triggerfish δ^34^S values were significantly higher than juvenile red snapper in the east (p = 0.001, Shaffer’s MCP) and central (p = 0.004, Shaffer’s MCP) regions. In contrast, adult gray triggerfish had significantly lower δ^34^S values than adult red snapper in the east region (p = 0.005, Shaffer’s MCP). No differences were observed between sub-adult gray triggerfish and red snapper δ^34^S values, which were similar across all three regions.

Gray triggerfish and red snapper δ^13^C and δ^15^N values generally decreased from east to west, while δ^34^S values increased across this gradient (Fig 4, S4 and S5 Tables). Sub-adult and adult gray triggerfish δ^13^C values were significantly higher in the east region compared to the central region (p < 0.0001, Shaffer’s MCP), while adult gray triggerfish δ^13^C values were higher in the east compared to the west region (Table 4, Fig 4A and 4B) (p < 0.0001, Shaffer’s MCP). There were no regional differences in juvenile gray triggerfish δ^13^C values (Table 4, Fig 4A). Sub-adult and adult red snapper δ^13^C values significantly differed across all three regions (east, central, west), and similar to gray triggerfish, δ^13^C values were highest in the east region and lowest in the west region (Fig 4D and 4E) (p < 0.0001, Shaffer’s MCP). Likewise, juvenile red snapper δ^13^C values were significantly lower in the west region compared to both the east and central regions (p < 0.0001, Shaffer’s MCP). Sub-adult gray triggerfish and red snapper had significantly higher δ^15^N values in the east region than in the central and west regions (p < 0.0001, Shaffer’s MCP). In addition, δ^15^N values for adult fishes (both species) significantly differed across all three regions (p < 0.0001, Shaffer’s MCP), with δ^15^N values decreasing from east to west for both species, while juvenile δ^15^N values did not differ by region for either species (Table 4, Fig 4A and 4D). Adult fishes for both species, and additionally sub-adult red snapper, had significantly lower δ^34^S values in the east region compared to the central and west regions (Table 4, Fig 4D and 4F) (p < 0.01, Shaffer’s MCP). Juvenile δ^34^S values did not differ by region for gray triggerfish, but were significantly higher in the west region compared to the east and central regions for red snapper (p < 0.0001, Shaffer’s MCP).

#### Isotopic niches

**Fig 5:**
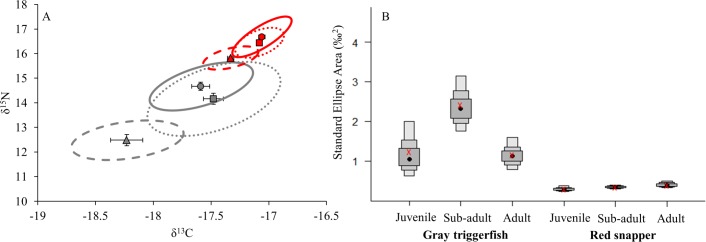
Species core isotopic niches and Bayesian credibles by size class, based on standard ellipse areas. (A) Standard ellipse areas contain 40% of the data for gray triggerfish (gray), and red snapper (red). Stable isotope means and ellipses (SEA_c_) are shown for juveniles (triangles, dashed ellipses), sub-adults (squares, dotted ellipses), and adults (circles, solid ellipses); error bars represent the standard error. (B) Bayesian credible intervals for gray triggerfish and red snapper standard ellipse areas by size class. Black points represent the mean, while gray boxes represent 50, 75, and 95% credible intervals. Red x’s represent SEA corrected for sample size (SEA_c_).

Isotopic niche size (SEA_c_) was generally similar among size classes (based on Bayesian credibles and bootstrapped confidence intervals) for both gray triggerfish and red snapper. Nonetheless, some differences in niche size were observed in gray triggerfish, as sub-adults had a larger SEA_c_ (2.42) than adults (1.15). Differences in niche metrics (CD, CR, NR, SR) were also minimal among size classes for both species; however red snapper adults exhibited a greater range of δ^15^N values compared to juveniles (Table 5 and Fig 5). Isotopic niche overlap between juvenile and sub-adult (gray triggerfish = 0.038, red snapper = 0.116), juvenile and adult (gray triggerfish = < 0.0001, red snapper = 0.102), and sub-adult and adult (gray triggerfish = 0.282, and red snapper = 0.365) size classes were non-significant (overlap < 0.6) for both species, indicating species-specific isotopic shifts among size classes. δ^13^C and/or δ^15^N values generally increased with size for both species, resulting in shifted ellipses by size class (Fig 5A). However, this shift was less distinct between sub-adult and adult fishes, as sub-adult gray triggerfish had a larger SEA_c_, and the overlap between sub-adults and adults for both species was much greater than observed among the other size classes.

**Table 5:** Niche metrics used for assessing species isotopic niches by size class, species, and region.

Analysis	SEA_c_	CD	δ^13^C Range		δ^15^N Range		δ^34^S Range	
			___________		__________		__________	
			Min	Max	Min	Max	Min	Max
*Species*								
**Juveniles**								
Gray triggerfish	1.24	0.75	-18.98	-17.33	10.99	14.18	17.17	20.00
Red snapper	0.30	0.40	-18.00	-16.87	14.35	16.94	18.05	19.83
**Sub-adults**								
Gray triggerfish	2.42	1.27	-18.58	-16.35	9.27	16.75	17.64	20.41
Red snapper	0.35	0.49	-18.06	-16.65	13.99	17.47	17.74	20.01
**Adults**								
Gray triggerfish	1.15	0.89	-18.35	-16.23	13.07	16.62	17.41	19.66
Red snapper	0.40	0.62	-18.09	-16.60	14.01	17.70	17.73	20.05
**East**								
Gray triggerfish	2.40	1.29	-18.61	-16.65	7.61	14.15	19.05	21.23
Red snapper	0.16	0.30	-17.84	-16.95	14.62	16.44	17.85	19.69
**Central**								
Gray triggerfish	1.22	0.91	-18.92	-17.08	8.83	15.07	18.78	21.80
Red snapper	0.19	0.34	-17.90	-17.01	13.72	15.90	18.02	19.68
**West**								
Gray triggerfish	0.93	0.71	-19.14	-17.51	9.83	13.13	17.88	21.70
Red snapper	0.62	1.01	-18.54	-17.35	12.37	16.18	19.68	20.27

The standard ellipse area (based on δ^13^C and δ^15^N) corrected for sample size (SEA_c_) is shown along with mean distance to centroid (CD), and δ^13^C, δ^15^N, and δ^34^S ranges (CR, NR, SR) for each species by size class and region.

**Fig 6:**
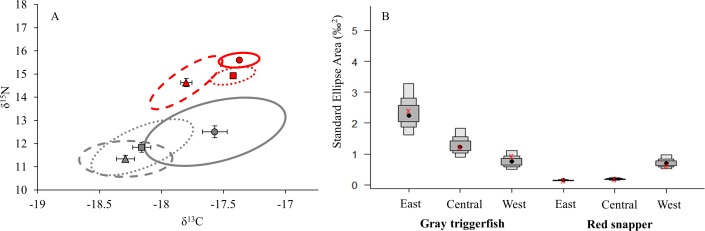
Species core isotopic niches and Bayesian credibles by region, based on standard ellipse areas. (A) Standard ellipse areas contain 40% of the data for gray triggerfish (gray), and red snapper (red). Stable isotope means and ellipses (SEA_c_) from length adjusted stable isotope values are shown for the east (circles, solid ellipses), central (squares, dotted ellipses), and west (triangles, dashed ellipses) regions; error bars represent the standard error. (B) Bayesian credible intervals for gray triggerfish and red snapper standard ellipse areas by region. Black points represent the mean, while gray boxes represent 50, 75, and 95% credible intervals. Red x’s represent SEA corrected for sample size (SEA_c_).

Isotopic niches and niche metrics differed between species regardless of size classes and regions, with exception of the west region (Table 5, Figs 5 and 6). Gray triggerfish had larger isotopic niches (SEA_c_) (Fig 5), with no significant overlap across all size classes relative to red snapper (overlap = 0.046 for juveniles, and < 0.0001 for sub-adults, adults). Similar trends were observed across regions, as gray triggerfish had larger isotopic niches than red snapper in the east and central regions, with no difference in niche size in the west region (Fig 6B). However, there was no significant overlap between gray triggerfish and red snapper isotopic niches in any of the three regions (overlap < 0.0001) (Fig 6). In addition, sub-adult gray triggerfish had wider ranging niche metrics (CD, CR, NR, and SR) compared to red snapper regardless of size class (Table 5). Similarly, niche metrics were wider ranging for gray triggerfish compared to red snapper in the east and central regions, with exception of SR (δ^34^S range), which was similar between species in the central region (Table 5). In contrast, species did not differ in the west region.

No significant overlap was observed among isotopic niches for either gray triggerfish or red snapper between the east and central (gray triggerfish = 0.136, red snapper = 0.002), east and west (gray triggerfish = 0.043, red snapper = 0.008), and central and west (gray triggerfish = 0.32, red snapper = 0.001) regions (Fig 6A). This trend reflects the general east to west decline in δ^13^C and/or δ^15^N values observed for both species (Fig 6A). Gray triggerfish isotopic niche size was smaller in the west region relative to the east; however, no differences in niche size were observed between the east and central regions or the central and west regions (Table 5, Fig 6). In contrast, isotopic nice size for red snapper was greatest in the west region, while no differences were observed between the east and central regions (Table 5, Fig 6). While gray triggerfish niche metrics (CD, CR, NR, SR) were similar across all three regions, red snapper from the west region exhibited greater trophic diversity (CD) and a larger range of δ^15^N values (NR). In addition, red snapper in the west region had a larger range of δ^34^S values (SR) compared to the central region.

#### Source contributions

**Fig 7:**
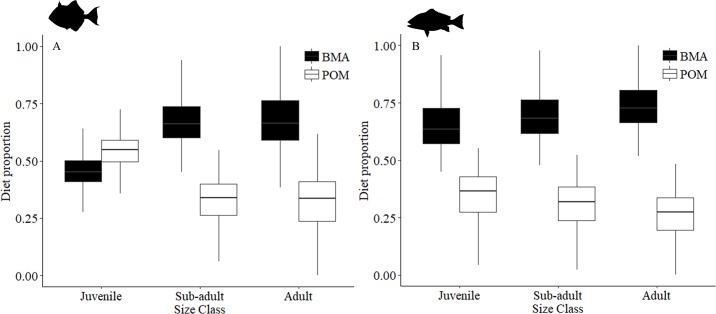
Pelagic (POM) and benthic (BMA) source contribution estimates for gray triggerfish and red snapper. Plots depict contribution estimates for POM and BMA to juvenile, sub-adult and adult size classes. (A) Depicts source contribution estimates for gray triggerfish, and (B) depicts source contribution estimates for red snapper.

Source contribution estimates from the Bayesian two-source mixing models were species specific and varied by size class. Pelagic and benthic carbon sources contributed to both gray triggerfish and red snapper (Fig 7). Contributions from benthic carbon were slightly higher than pelagic for both species across all size classes, juveniles (gray triggerfish: 47 ± 8.6% SD, red snapper: 66 ± 11.0% SD), sub-adults (gray triggerfish: 68 ± 10.0% SD, red snapper: 70 ± 14.0% SD), and adults (gray triggerfish: 68 ± 12.0% SD, red snapper: 74 ± 10.0% SD), except for juvenile gray triggerfish, for which pelagic contribution estimates were higher (54 ± 8.6% SD). Benthic carbon contribution increased with size for both species, increasing by ~21% from juveniles for sub-adult and adult gray triggerfish and ~4% and ~8% from juveniles for sub-adult and adult red snapper, respectively. In addition, gray triggerfish had slightly higher contributions from pelagic sources than red snapper across all size classes (mean difference = 9%). It should be noted that pelagic and benthic sources were negatively correlated in the diagnostic matrix plots of the posterior distributions for the models, which may be indicative of a missing source/primary producer associated with artificial reefs, such as red algae, green algae, and epiphytes [15].

## Discussion

Gray triggerfish and red snapper demonstrated diverse diets at artificial reefs in the northwest GoM, with 66 prey taxa identified in gray triggerfish and 47 in red snapper, supporting the notion that these species are generalists predators [7, 20]. The two species generally increased in trophic position with size and consumed similar prey groups; however, ontogenetic trends in stable isotopes and relative contributions (%IRI and % contributions) of taxa within these prey groups differed by size class, species, and region, indicating ontogenetic as well as species and region-specific differences in foraging. While crabs were an important prey group for both species, xanthid crabs were more commonly consumed by gray triggerfish, and portunid crabs were predominately consumed by red snapper. Similarly, the prominence of bivalves and pelagic gastropods in the diets of gray triggerfish relative to red snapper suggests differences in foraging behavior, where gray triggerfish diets encompassed a greater diversity of prey. Lastly, regional differences in feeding suggested differences in prey availability among regions, as crabs were the dominant prey in the east region and gastropod consumption increased from east to west.

Gray triggerfish diets were similar to reports from the southeastern United States identifying gastropods, decapods, bivalves, and barnacles as primary prey groups [7, 67]. Likewise, gray triggerfish are known to consume large numbers of pelagic gastropods (pteropods) [7], which is in agreement with the current study, where the majority of identified gastropods in gray triggerfish guts were pelagic taxa (e.g. Cavolinidae, Atlantidae, and Limacinidae). In the GoM, reports on gray triggerfish feeding consist of observations of sand dollar predation [38, 39, 67], which were absent in the gut contents of this study, possibly due to differences in sand dollar abundance, as the previous studies were conducted off the Florida coast. For red snapper, the predominance of stomatopods and fishes is in accord with other studies in the GoM examining similar sized individuals [20, 23, 33]. However, this study differed from others in that squid were not a primary contributor to the diet. Because red snapper are highly opportunistic foragers [20], this may be due to seasonal or regional differences in local prey abundances at our sites in comparison to those sampled in other studies. Reports of squid as a prominent prey group in red snapper [23, 33] included fall and winter sampling, while this study focused around spring and summer months. Indeed, Wells et al. [23] found squid to be more important in the fall and winter, while fishes were more prominent during the summer.

Ontogenetic shifts in diet were observed in the gut contents of gray triggerfish when examined by size class, while ontogenetic shifts for snapper were not evident. Juvenile gray triggerfish diets constituted a greater proportion of crabs (xanthid) compared to sub-adult and adult fish, which may reflect an affinity for the bottom or structure at smaller sizes, due to increased refuge from predators [68]. Nevertheless, sample size was small for juvenile gray triggerfish (n = 13), and may not have been fully representative of the diet. Studies examining ontogenetic shifts in diet for gray triggerfish are limited, but a study off the southeastern United States examining similar sized fish found that individuals < 400 mm TL primarily consumed decapod shrimp while individuals > 400 mm TL consumed a greater proportion of barnacles and bivalves [7]. In contrast, gray triggerfish in the current study ranged from 111 to 382 mm FL, and while shrimp were identified in the gut contents, they constituted less than 1% of the contents by weight. While no ontogenetic shifts in diet were observed in red snapper gut contents, changes in diet with ontogeny have been reported in other studies that encompassed a wider size range, including newly settled individuals < 60 mm TL [23, 33]. Red snapper in this study ranged from 151 to 612 mm TL, thus newly settled juveniles were not included in the sample.

Sessile taxa associated with hard substrate were more commonly consumed by gray triggerfish at all size classes, with several such taxa unique to their diet. While gray triggerfish and red snapper consumed both sessile and mobile prey, the greater diversity and proportion of sessile organisms (i.e. reef-attached; barnacles, bivalves: Mytilidae, Plicatulidae, Pteriidae, Chamidae, and Campanulariidae) in gray triggerfish guts indicates more frequent foraging on the reef structure, as gray triggerfish have been shown to remain close to reefs (mean distance = 35.9 m), with relatively high site fidelity and residency (> 1 year) [69]. Also, gray triggerfish possess unique dentition and jaw morphology that is suitable for consuming hard-bodied sessile organisms [67], which possibly enable greater feeding opportunities on reef-attached organisms compared to red snapper. While red snapper also fed on bivalves, they contributed relatively little to the overall diet (0.63 ± 0.67% to 2.42 ± 1.32%). While gastropods were generally more important to the diet of gray triggerfish than red snapper, gastropods were consumed in similar amounts by both species in the west region. This could be a result of differences in prey availability among regions, as both species also had greater % contributions from fish and lower % contributions from crabs in the west region. With the exception of adult gray triggerfish and red snapper (similar contribution of fish to the diet), consumption of stomatopods, fishes, and portunid crabs was greater across all size classes and regions for red snapper, suggesting that red snapper may depend more on non-reef prey associated with open mud and/or sand bottom [23, 33] surrounding artificial reefs.

Gray triggerfish and red snapper muscle tissue δ^13^C values were comparable to those previously reported in the GoM, while δ^15^N values were slightly higher. Reported δ^13^C values for gray triggerfish muscle tissue at artificial reefs are limited, but values based on small sample sizes collected at oil and gas platforms off the Louisiana coast (n = 4, -17.83 ‰) [15] were similar to results of the current study (-17.62 ‰). Likewise, red snapper δ^13^C values (-17.13 ‰) were similar to those previously reported at artificial reefs (oil and gas platforms in addition to non-platform reefs such as cement blocks and wrecks) off the Texas coast [34]. While the current study utilized lipid extraction before obtaining δ^13^C values, and reports for gray triggerfish [15] and red snapper [34] δ^13^C values elsewhere in the GoM did not, lipid extraction for tissues with low lipid concentrations (such as animal muscle tissue) has little impact on δ^13^C values [70]. δ^15^N values for red snapper (16.37 ‰) were higher compared to those at artificial reefs in the northeast GoM (~15 ‰); however, these studies found significant contribution from lower trophic level prey (zooplankton) to red snapper diets [20], which were not major contributors to the diets in this study and likely resulted in higher δ^15^N values. Species δ^34^S values were comparable to consumers in other marine systems (16–18 ‰) where the substrate (course and fine sands) was similar to that surrounding the reef sites in the current study [71].

Increases in δ^13^C and δ^15^N values with size class for gray triggerfish and red snapper were consistent with studies examining ontogenetic shifts in diet [23, 33]. This pattern is well documented, as rapid increases in body size enables fish to consume a greater diversity of prey items, especially in the first few years of life when growth is accelerated [23, 59]. Higher δ^34^S values for juvenile gray triggerfish in the east region and juvenile red snapper in the central region is likely reflective of greater contribution from pelagic carbon to juveniles in these regions. Conversely, decreased δ^15^N values between sub-adult and adult red snapper in the west region suggests that red snapper consumed a greater proportion of lower trophic level prey with increasing size in this region. This may reflect regional prey availability, as gastropod consumption, solely consisting of pelagic species from family Cavolinidae, was substantially greater in the west compared to the east and central regions. A similar inverse relationship between size and trophic level was described for red snapper in the northeast GoM, where fish > 500 mm consumed a greater proportion of zooplankton compared to smaller sized fish [20], thus regional or local prey availability likely effects species-specific ontogenetic dietary shifts.

Gray triggerfish exhibited lower δ^13^C and δ^15^N values relative to red snapper across all size classes and regions, which may be due to more frequent foraging on lower trophic level prey, such as filter feeding benthic invertebrates (bivalves) and pteropods. This is supported by higher δ^34^S values for gray triggerfish, which suggest a greater contribution from pelagic carbon. Interestingly, adult gray triggerfish in the east region had lower δ^34^S values than adult red snapper, which is likely due to differences in prey availability across the three regions, as both species consumed a greater proportion of benthic prey such as crabs and substantially less pelagic gastropods in this region. In addition, red snapper generally consumed more fish and less invertebrate prey (bivalves, barnacles, pteropods etc.) than gray triggerfish, and would be expected to occupy a higher trophic position, as species that consume large amounts of fish generally have higher δ^15^N values than species primarily consuming invertebrate prey [72].

Size-specific isotopic niches and niche metrics (CD, CR, NR, SR) indicated diet diversity was similar across most size classes for each species. While not evident in the gut content analysis, isotopic niche analyses indicated that sub-adult gray triggerfish had a more diverse diet (larger isotopic niche) compared to adults, which likely reflects larger fish specializing on higher trophic level prey, such as fish [72]. Like adult gray triggerfish, adult red snapper likely consume a greater proportion of higher trophic level prey (i.e. fish); however a greater range of δ ^15^N values suggests that they also consume a high proportion of lower trophic level prey (crabs, stomatopods, pteropods) items that dominate the diets of smaller (juvenile and sub-adult) fish. However, this finding may be heavily influenced by the differences in ontogeny observed for red snapper in the west region, as we were unable to assess species isotopic niches and niche metrics by size class within region. Lastly, isotopic shifts among size classes corroborated results from the stable isotope analyses (MANOVA, ANOVA), where generally increasing δ^15^N values with size indicated increasing trophic position.

Gray triggerfish had larger isotopic niches across all size classes and most regions (niche size was similar between species in the west), suggesting a more diverse diet, encompassing a greater variety of pelagic and benthic prey (as evidenced by the gut content analysis). This finding is consistent with the greater number of taxa identified in gray triggerfish gut contents relative to red snapper, as well as other studies that describe gray triggerfish as a flexible forager with a wide niche breadth [7, 67, 73]. Interestingly, despite similar contribution estimates for crabs in species’ diets, no significant overlap was observed (including the west region). This is likely due to family level taxonomic differences in diet not accounted for in the broader prey categories, such as the greater proportion of xanthid crabs in gray triggerfish and portunid crabs in red snapper [20, 33, 67, 74]. In contrast, gray triggerfish and red snapper isotopic niches were similar in size in the west region, which is likely explained by the increased gastropod consumption and lower δ^15^N values observed for red snapper in the west compared to the east and central regions.

Source contribution estimates are important for understanding energy flow and identifying essential resources to consumers at artificial reefs. Stable isotope values of POM and BMA were comparable to previously reported values in the GoM (δ^13^C = 19–22 ‰ and δ ^15^N = 5–7 ‰ for POM; δ^13^C = 14.7–19.9 ‰ and δ ^15^N = 6.7–7.8 ‰ for BMA) [15, 23, 66]. Gray triggerfish and red snapper had significant contributions from both pelagic and benthic sources; however, benthic contribution estimates were greater for both species within all size classes (except juvenile gray triggerfish), suggesting that benthic primary production may be important for consumers at artificial reefs. In contrast, juvenile gray triggerfish spend early life in pelagic sargassum and recruit to benthic habitats much later (4–7 months [75]) than red snapper (~30 days [76]), and are thus more likely to reflect feeding in the pelagic environment due to limited time for tissue turnover, which may occur on the scale of weeks to months [77]. While our results show the importance of both pelagic and benthic carbon to gray triggerfish, pelagic contribution estimates presented here (~39%) were similar to those estimated from a small sample of individuals (n = 4) at offshore oil and gas platforms (~37% [15]). Nevertheless, because previous estimates of source contributions for adult gray triggerfish are limited (estimates of POM for 4 individuals [15]), this study represents the most robust estimates to date for the contribution of POM and BMA to this species.

Increased influence of benthic primary productivity with ontogeny for gray triggerfish and red snapper supports findings from Wells et al. [23] that demonstrated increases in benthic contributions to red snapper with age (34–51% from age 1–3 [23]), suggesting that both species may become more dependent on benthic sources with age and as reef association increases [75, 78]. Despite the importance of benthic carbon to older fish of both species, pelagic contribution was slightly higher for gray triggerfish at all size classes, which likely reflects the importance of filter feeding invertebrates to the diet of this species. Although mixing model diagnostics indicated a potential missing source, a study examining the importance of phytoplankton, macroalgae (red and green algae), and epiphytes as sources of primary production at an oil platform found phytoplankton-derived organic matter to be the dominant resource for all consumers examined [15], suggesting that macroalgae and epiphytes on the reef structure may not play as large of a role to the feeding ecology of reef-associated fauna.

Pronounced regional differences in gray triggerfish and red snapper diets were likely caused by a combination of environmental factors that differed among regions (depth, structure, physiochemical properties), and are known to affect reef fish community structure and foraging [29, 68, 79, 80]. Although we were unable to test these factors independently of one another, reef material and complexity varied across the three regions and likely played a significant role in reef fish demographics and prey community composition [29, 68, 79]. Reef sites in the west region contained complex structures (ships) not found in the other regions, which may attract a greater number of fishes [18] and increase forage species diversity and richness [68]. In addition, it is possible that regional differences in freshwater inflow may have influenced prey communities and the isotopic signatures of prey targeted by gray triggerfish and red snapper.

Though water parameters were relatively similar during the season in which we sampled (summer), an east to west salinity gradient exists along the Texas coast that is present for much of the year [40]. While we did not collect prey at our sites, inshore fishes (Ariidae, Sciaenidae), characteristic of lower salinities were more commonly caught as bycatch in the east and central regions, while more diverse reef fish communities (e.g., Carangidae, Serranidae, and Lutjanidae) were observed at the more complex sites in the west region. In addition, there is significant variability in fresh water influx from the Mississippi river in this region, which can result in seasonal variations in source (POM and BMA) stable isotope values [81]. Because sampling for this work primarily took place in the summer, such seasonal variations in source were not accounted for.

The greater proportion of crabs consumed by both species in the east region and increasing gastropod consumption in the central and west regions suggests regional differences in prey abundances may be reflected in the diets of both species. Interestingly, this trend had differing effects on species isotopic niches, as gray triggerfish had a larger isotopic niche in the east region and red snapper had a larger isotopic niche in the west, indicating that gray triggerfish diets were more diverse when gastropods constituted a relatively small proportion of the diet and red snapper diets were more diverse when gastropods constituted a high proportion of the diet. In addition, regional differences in prey communities could explain the differing ontogenetic trend in δ^15^N for red snapper in the west (decreasing δ^15^N values with age) as the contribution from lower trophic level prey, such as pelagic gastropods, was highest in this region. In high abundance, these lower trophic level prey groups may become a reliable food source with low energetic cost for larger individuals to meet their dietary requirements. Similar decreasing δ^15^N values with age have been observed for red snapper in the northeast GoM, where zooplankton consumption increased with size [20]. Likewise, generally higher δ^34^S values for red snapper in the west also supports a shift to more pelagic feeding.

This study examined the trophic interactions and feeding ecology of two of the more common reef fish species at artificial reefs in the northwest GoM. Results highlight the importance of pelagic and benthic primary production to upper-level consumers at these artificial reefs, and demonstrate that gray triggerfish and red snapper exhibit some degree of resource partitioning. Although gray triggerfish feed on benthic (bivalves, crabs) and pelagic (pteropods) prey, the diet of this species was more dependent on organisms associated with the artificial reef structure. Red snapper occupied a higher trophic position than gray triggerfish, and consumed prey primarily associated with the surrounding substrate, which suggests less direct dependence on the artificial reef for foraging opportunities. Regional differences in gut contents and stable isotope values (decreasing δ^13^C and δ^15^N values from east to west), and isotopic niches for both species were likely reflective of difference in prey availability associated with environmental variability (reef structure, depth, physiochemical properties of seawater) among the three regions, and support the notion that both species are generalist predators. Although the effects of depth and reef structure were not determined in this study, artificial reef literature and regional site characteristics suggest that prey communities may have differed across our study area, contributing to the observed differences in regional gut contents and stable isotopes. Findings here highlight the importance of pelagic primary production to higher-level consumers at artificial reefs [23, 82], and provide additional support on the role of BMA as a carbon source to fishes utilizing nearshore artificial reefs.

## Supporting information

S1 Table Permutational analysis of variance examining gray triggerfish and red snapper gut contents. Results are shown from PERMANOVA examining prey group composition by size class, species, and region; a ‘*’ indicates a significant result.
(PDF)

S2 Table Percent weight (%W) of constituent taxa (family level) for primary prey groups by size class. Prey groups in bold are those that contributed most to the dissimilarity in gut contents between species and among size classes and regions. Numbers in bold represent the total %W for all the taxa within a prey group (i.e. crabs).
(PDF)

S3 Table Percent weight (%W) of constituent taxa (family level) for primary prey groups by region. Prey groups in bold are those that contributed most to the dissimilarity in gut contents between species and among size classes and regions. Numbers in bold represent the total %W for all the taxa within a prey group (i.e. crabs).
(PDF)

S4 Table Multivariate analysis of variance (MANOVA) examining differences in δ^13^C, δ^15^N, and δ^34^S. Differences in δ^13^C, δ^15^N, and δ^34^S by species, size class, and region were examined. A total of 89 gray triggerfish and 327 red snapper were analyzed. A ‘*’ indicates significant results.
(PDF)

S5 Table Analysis of variance (ANOVA) for δ^13^C, δ^15^N, and δ^34^S. Differences in δ^13^C, δ^15^N, and δ^34^S by species, size class, and region were examined. A total of 89 gray triggerfish and 327 red snapper were analyzed. A ‘*’ indicates significant results.
(PDF)
