# Supplementary material for: Feeding ecology of fishes associated with artificial reefs in the northwest Gulf of Mexico
Source: PLoS One. 2018 Oct 2;13(10):e0203873. doi: 10.1371/journal.pone.0203873 (PMC6168147; doi:10.1371/journal.pone.0203873)
Supplement: S1 Table — Results are shown from PERMANOVA examining prey group composition by size class, species, and region; a ‘*’ indicates a significant result. (PDF) [file pone.0203873.s001.pdf]

**Factor**	**df**	**SS**	**MS**	**Pseudo-F**	**p-value**	**Unique perms**
Species	1	31484	31484	14.136	0.001*	998
Size class	2	9812.3	4906.1	2.203	0.026*	998
Region	2	42717	21358	9.590	0.001*	998
Species x size class	2	10217	5108.5	2.294	0.021*	999
Species x region	2	17299	8649.5	3.884	0.001*	999
Size class x region	4	11338	2834.6	1.273	0.198	998
Species x size class x region	4	14883	3720.7	1.671	0.057	997
