# Supplementary material for: Feeding ecology of fishes associated with artificial reefs in the northwest Gulf of Mexico
Source: PLoS One. 2018 Oct 2;13(10):e0203873. doi: 10.1371/journal.pone.0203873 (PMC6168147; doi:10.1371/journal.pone.0203873)
Supplement: S2 Table — Prey groups in bold are those that contributed most to the dissimilarity in gut contents between species and among size classes and regions. Numbers in bold represent the total %W for all the taxa within a prey group (i.e. crabs). (PDF) [file pone.0203873.s002.pdf]

Prey	Gray triggerfish			Red snapper		
	Juveniles	Sub-adults	Adults	Juveniles	Sub-adults	Adults
**Crabs**	**41.67**	**19.15**	**17.85**	**18.02**	**33.39**	**28.17**
unknown crabs	1.24	3.49	2.20	9.46	11.93	10.96
Porcellanidae	0.00	0.14	0.07	0.62	0.00	0.00
Paguroidea	0.00	0.00	0.62	0.00	0.00	0.00
Hepatidae	0.00	0.05	2.30	0.00	4.19	0.97
Leucosiidae	0.00	0.39	0.47	1.61	1.25	0.00
Portunidae	0.00	0.84	3.26	1.77	10.97	15.91
Xanthoidea	40.44	14.25	8.94	4.56	5.06	0.33
**Bivalves**	**7.94**	**23.40**	**10.57**	**1.79**	**2.28**	**0.01**
unknown bivalves	2.43	2.58	0.78	0.86	2.26	0.01
Arcidae	1.16	12.58	2.09	0.13	0.00	0.00
Crassatellidae	0.00	0.00	0.00	0.59	0.00	0.00
Corbulidae	0.00	0.00	0.00	0.20	0.00	0.00
Mytilidae	3.96	7.82	6.15	0.00	0.00	0.00
Nuclanidae	0.00	0.00	0.00	0.00	0.02	0.00
Plicatulidae	0.00	0.21	0.00	0.00	0.00	0.00
Pteriidae	0.00	0.03	0.00	0.00	0.00	0.00
Chamidae	0.38	0.18	1.05	0.00	0.00	0.00
Veneridae	0.00	0.00	0.50	0.00	0.00	0.00
**Fishes**	**0.05**	**1.92**	**12.56**	**15.56**	**7.15**	**8.95**
unknown fish	0.05	1.92	12.56	13.54	6.69	8.95
Syngnathidae	0.00	0.00	0.00	0.00	0.00	0.00
Sciaenidae	0.00	0.00	0.00	0.00	0.46	0.00
Blenniidae	0.00	0.00	0.00	2.02	0.00	0.00
**Gastropods**	**1.57**	**9.47**	**9.42**	**0.49**	**0.14**	**0.18**
unknown gastropods	1.56	0.58	1.57	0.00	0.01	0.00
Atlantidae	0.00	0.00	0.04	0.00	0.00	0.00
Collumbellidae	0.01	0.00	0.00	0.02	0.00	0.00
Nassariidae	0.00	0.00	0.04	0.00	0.02	0.00
Fissurellidae	0.00	0.00	0.11	0.00	0.00	0.00
Limacinidae	0.00	0.02	0.00	0.00	0.00	0.00
Natcidae	0.00	0.02	0.08	0.07	0.00	0.00
Pyramidellidae	0.00	0.00	0.00	0.00	0.00	0.00
Hipponicidae	0.00	0.00	0.14	0.00	0.00	0.00
Cavolinidae	0.00	8.81	7.43	0.40	0.11	0.18
Lottidae	0.00	0.03	0.00	0.00	0.00	0.00
**Stomatopods**	**0.00**	**0.00**	**0.21**	**12.84**	**9.43**	**2.72**
Squillidae	0.00	0.00	0.21	12.84	9.43	2.72
