# Supplementary material for: Feeding ecology of fishes associated with artificial reefs in the northwest Gulf of Mexico
Source: PLoS One. 2018 Oct 2;13(10):e0203873. doi: 10.1371/journal.pone.0203873 (PMC6168147; doi:10.1371/journal.pone.0203873)
Supplement: S3 Table — Prey groups in bold are those that contributed most to the dissimilarity in gut contents between species and among size classes and regions. Numbers in bold represent the total %W for all the taxa within a prey group (i.e. crabs). (PDF) [file pone.0203873.s003.pdf]

**Prey**	**Gray triggerfish**			**Red snapper**		
	East	Central	West	East	Central	West
**Crabs**	**19.36**	**23.14**	**14.17**	**32.84**	**13.31**	**4.76**
unknown crabs	5.88	0.36	0.16	12.13	7.44	1.36
Porcellanidae	0.21	0.00	0.00	0.04	0.00	0.00
Paguroidea	0.00	0.00	1.44	0.00	0.00	0.00
Hepatidae	2.18	0.00	0.00	2.48	0.00	0.00
Leucosiidae	0.96	0.00	0.00	0.58	0.38	0.00
Portunidae	4.91	0.01	0.00	15.18	4.57	0.00
Xanthoidea	5.22	22.77	12.56	2.44	0.92	3.40
**Bivalves**	**20.81**	**20.44**	**0.82**	**1.07**	**0.13**	**0.05**
unknown bivalves	3.12	0.47	0.49	1.02	0.05	0.05
Arcidae	14.64	0.08	0.09	0.01	0.00	0.00
Crassatellidae	0.00	0.00	0.00	0.04	0.00	0.00
Corbulidae	0.00	0.00	0.00	0.00	0.08	0.00
Mytilidae	2.12	16.90	0.00	0.00	0.00	0.00
Nuclanidae	0.00	0.00	0.00	0.01	0.00	0.00
Plicatulidae	0.20	0.00	0.00	0.00	0.00	0.00
Pteriidae	0.10	1.24	0.00	0.00	0.00	0.00
Chamidae	0.00	1.76	0.25	0.00	0.00	0.00
Veneridae	0.63	0.00	0.00	0.00	0.00	0.00
**Fishes**	**10.83**	**1.55**	**10.31**	**8.29**	**9.22**	**17.06**
unknown fish	10.83	1.55	10.31	8.09	9.22	13.01
Syngnathidae	0.00	0.00	0.00	0.00	0.00	0.00
Sciaenidae	0.00	0.00	0.00	0.20	0.00	0.00
Blenniidae	0.00	0.00	0.00	0.00	0.00	4.05
**Gastropods**	**0.23**	**18.88**	**10.38**	**0.13**	**0.25**	**1.70**
unknown gastropods	0.00	0.93	3.67	0.00	0.01	0.00
Atlantidae	0.00	0.03	0.05	0.00	0.00	0.00
Collumbellidae	0.05	0.00	0.00	0.00	0.00	0.00
Nassariidae	0.00	0.00	0.00	0.01	0.00	0.00
Fissurellidae	0.00	0.18	0.00	0.00	0.00	0.00
Limacinidae	0.02	0.00	0.00	0.00	0.00	0.00
Natcidae	0.00	0.02	0.21	0.00	0.01	0.00
Pyramidellidae	0.00	0.00	0.00	0.00	0.00	0.00
Hipponicidae	0.00	0.22	0.00	0.00	0.00	0.00
Cavolinidae	0.16	17.46	6.45	0.11	0.23	1.70
Lottidae	0.00	0.04	0.00	0.00	0.00	0.00
**Stomatopods**	**0.27**	**0.00**	**0.00**	**4.04**	**34.86**	**13.99**
Squillidae	0.27	0.00	0.00	4.04	34.86	13.99
