# Supplementary material for: Feeding ecology of fishes associated with artificial reefs in the northwest Gulf of Mexico
Source: PLoS One. 2018 Oct 2;13(10):e0203873. doi: 10.1371/journal.pone.0203873 (PMC6168147; doi:10.1371/journal.pone.0203873)
Supplement: S4 Table — Differences in δ13C, δ15N, and δ34S by species, size class, and region were examined. A total of 89 gray triggerfish and 327 red snapper were analyzed. A ‘*’ indicates significant results. (PDF) [file pone.0203873.s004.pdf]

Factor	num df	den df	*F*-value	p-value
**MANOVA: ($\delta^{13}C$, $\delta^{15}N$, $\delta^{34}S$)**				
Species	3	396	335.53	< 0.0001*
Size class	6	794	30.80	< 0.0001*
Region	6	794	31.17	< 0.0001*
Species x size class	6	794	8.63	< 0.0001*
Species x region	6	794	11.55	< 0.0001*
Size class x region	12	1194	4.80	< 0.0001*
Species x size class x region	12	1194	3.76	< 0.0001*
