# Supplementary material for: Feeding ecology of fishes associated with artificial reefs in the northwest Gulf of Mexico
Source: PLoS One. 2018 Oct 2;13(10):e0203873. doi: 10.1371/journal.pone.0203873 (PMC6168147; doi:10.1371/journal.pone.0203873)
Supplement: S5 Table — Differences in δ13C, δ15N, and δ34S by species, size class, and region were examined. A total of 89 gray triggerfish and 327 red snapper were analyzed. A ‘*’ indicates significant results. (PDF) [file pone.0203873.s005.pdf]

**Factor**	**df**	***F*-value**	**p-value**
**ANOVA: $\delta^{13}C$**			
Species	1	270.03	< 0.0001*
Size class	2	56.29	< 0.0001*
Region	2	78.95	< 0.0001*
Species x size class	2	13.06	< 0.0001*
Species x region	2	27.54	< 0.0001*
Size class x region	4	2.45	0.046*
Species x size class x region	4	5.49	0.001*
**ANOVA: $\delta^{15}N$**			
Species	1	817.39	< 0.0001*
Size class	2	67.71	< 0.0001*
Region	2	71.43	< 0.0001*
Species x size class	2	20.55	< 0.0001*
Species x region	2	0.42	0.656
Size class x region	4	13.15	< 0.0001*
Species x size class x region	4	1.31	0.265
**ANOVA: $\delta^{34}S$**			
Species	1	2.71	0.0001*
Size class	2	1.24	0.001*
Region	2	7.03	< 0.0001*
Species x size class	2	0.77	0.013*
Species x region	2	0.51	0.056
Size class x region	4	0.47	0.033*
Species x size class x region	4	0.54	0.017*
